# Inhibitory activity of metal-curcumin complexes on quorum sensing related virulence factors of *Pseudomonas aeruginosa* PAO1

**DOI:** 10.1186/s13568-020-01045-z

**Published:** 2020-06-08

**Authors:** Mina Gholami, Habib Zeighami, Rahman Bikas, Azam Heidari, Farzaneh Rafiee, Fakhri Haghi

**Affiliations:** 1 Department of Microbiology, School of Medicine, Zanjan University of Medical Sciences, Zanjan, Iran; 2 Department of Chemistry, Faculty of Science, Imam Khomeini International University, 34148-96818 Qazvin, Iran; 3 School of Medicine, Zanjan University of Medical Sciences, Zanjan, Iran

**Keywords:** Metal–curcumin complex, *Pseudomonas aeruginosa*, Quorum sensing, Virulence factor

## Abstract

The use of metal complexes to reduce or inhibit virulence factors of *Pseudomonas aeruginosa* is a promising strategy for the management and control of infections caused by this multidrug-resistant pathogen. The present study aimed to investigate the anti-quorum sensing activity of sub-minimum inhibitory concentrations (sub-MIC) of copper(II) sulfate pentahydrate-curcumin complex (Cu-CUR), iron(III) nitrate nonahydrate -curcumin complex (Fe-CUR), zinc(II) chloride-curcumin complex (Zn-CUR) and free curcumin (free-CUR) against *P. aeruginosa* PAO1. Metal-CUR complexes were synthesized and characterized by spectroscopic methods. The effect of sub-MIC (1/4 and 1/16 MIC) concentrations of metal-CUR complexes and free-CUR on cell growth, biofilm formation, motility, alginate and pyocyanin production, H_2_O_2_ susceptibility and expression of *lasI* and *lasR* genes in PAO1 was determined. MIC of metal-CUR complexes and free-CUR was determined as 62.5 and 125 µg/ml, respectively. Metal-CUR complexes at concentration of 62.5 µg/ml significantly reduced the cell growth to 1.5%–3.3%. Although we did not measure the anti-QS activity of metal-CUR complexes directly against PAO1, they indicated anti-QS activity in *C. violaceum* CV026. Copper-CUR complex at the concentration of 1/4 MIC showed the greatest inhibitory effect on swarming and twitching motilities, biofilm formation, alginate and pyocyanin production, sensitivity to H_2_O_2_ and reduction in the expression levels of *lasI* and *lasR* genes (P < 0.001). Considering the biological effects of Cu-CUR complex and its inhibitory activity on virulence factors, it may be used as an effective compound for treatment and control of infections caused by *P. aeruginosa*.

## Introduction

Quorum sensing (QS) as a key two component bacterial system plays an important role in controlling of bacterial virulence factors. In the quorum sensing process, in response to the presence of small signal molecules called autoinducers (AIs), bacteria regulate the expression of certain genes (Abisado et al. 2018). *Pseudomonas aeruginosa*, as an opportunistic and nosocomial human pathogen, uses this system to control its virulence factors. QS in *P. aeruginosa* consists of four systems including LasI/LasR, RhlI/RhlR and PQS/MvfR and IQS (Lee and Zhang 2015; Sarabhai et al. 2016). Inhibition of QS in drug resistant bacteria is considered as an effective strategy for development of antipathogenic agents and control of microbial infections. Therefore, quorum sensing inhibitors (QSIs) can be used to attenuate the virulence and pathogenesis and may have a role in control and treatment of acute and persistent infections (Rémy et al. 2018). The QSIs can be inhibiting the signal generator, degrade the signal molecule or blockage the signal receptor. In recent years, several natural and chemically synthesized quorum sensing inhibitors have been reported (Defoirdt et al. 2013, Koh et al. 2013). Curcumin or diferuloylmethane with chemical formula of (1,7-bis (4-hydroxy-3-methoxyphenyl)-1,6-heptadiene-3,5-dione) is a natural component of the *Curcuma longa* (turmeric) rhizome. Several investigations have reported the broad-spectrum anti-bacterial, anti-viral and anti-fungal activities of curcumin. It also has anti-oxidant, anti-inflammatory and anti-cancer effects and has a potential against various diseases such as diabetes, allergies, arthritis, Alzheimer’s and other chronic diseases (Moghadamtousi et al. 2014; Tyagi et al. 2015). Metal complexes are promising agents for improvement of antimicrobial activity. It has been reported that the metal complexes possess more biological and antimicrobial activities against Gram-negative and Gram-positive bacteria than free ligands (Behera et al. 2012; Wang et al. 2014; Ghosh et al. 2015; Meza-Morales et al. 2019a). The most important variables affecting antibacterial activity of metal complexes include chemistry, particle size, particle shape and zeta potential. Zeta potential plays a significant role in the ability of metal particles to penetrate into cell (Seil and Webster 2012). Metal complexes target several cellular processes leading to pleiotropic effects on bacterial cells; while antibiotics affect specific biochemical processes. The main purpose of the development of new antimicrobial agents is to achieve high efficacy at low doses without the evolution of resistance Metal complexes are probably to evolve less resistance (Turner 2017). Previous studies have been carried out on the antibacterial activities of some metal-curcumin complexes but the effect of these compounds on *P. aeruginosa* QS system has not been demonstrated (Bagchi et al. 2015; Syed et al. 2015; Girish et al. 2019; Tran Quang and Thao 2019). So, the present study aimed to investigate the anti-quorum sensing activity of sub-MIC concentrations of copper(II) sulfate pentahydrate (CuSO_4_·5H_2_O)-curcumin complex (Cu-CUR), iron(III) nitrate nonahydrate (Fe(NO_3_)_3_·9H_2_O)-curcumin complex (Fe-CUR), zinc(II) chloride (ZnCl_2_)-curcumin complex (Zn-CUR) and free curcumin (free-CUR) against *P. aeruginosa* PAO1.

## Materials and methods

### Materials and instrumentation

Curcumin, copper(II) sulfate pentahydrate (CuSO_4_·5H_2_O), zinc(II) chloride (ZnCl_2_) and iron(III) nitrate nonahydrate (Fe(NO_3_)_3_·9H_2_O) were purchased from Merck (Germany). Solvents with highest purity were bought from Merck (Germany) and used without further purifications. The elemental analyses (carbon, hydrogen and nitrogen) of complexes were obtained from a Carlo ERBA Model EA 1108 analyzer. The metal content of the complexes was determined by atomic absorption analysis on a Varian Spectra AA-220 equipment. Fourier transform infrared (FT-IR) spectroscopy was performed using a FT-IR Spectrometer Bruker Tensor 27 as KBr disks. Fresh stock solution of metal-CUR complexes (Cu-CUR, Zn-CUR, Fe-CUR) and free-CUR were prepared in dimethyl sulfoxide (DMSO) at the concentration of 1 mg/ml. N-Acyl-homoserine lactone (C6-HSL) (Sigma-Aldrich) was used at 20 µM in *C. violaceum* CV026 biosensor bioassay.

### Bacterial strains, growth media and conditions

To assay the anti-quorum sensing activity of metal-CUR complexes (Cu-CUR, Zn-CUR, Fe-CUR) and free-CUR, wild type *P. aeruginosa* PAO_1_ and reporter strain *of Chromobacterium violaceum* CV026 (Gift given by Dr Hassan Rokni-zadeh, Department of Biotechnology, Zanjan University of Medical Sciences, Zanjan, Iran) were used. The cultures were grown in Lysogeny broth/agar (Merck, Germany) aerobically at 37 °C or 30 °C for 16–18 h. Strains were preserved at − 80 °C in Tryptic Soy Broth (TSB, Merck, Germany) containing 10% (v/v) glycerol.

### Synthesis of metal-curcumin complexes

#### Synthesis of Cu-CUR

The Cu-CUR complex was synthesized by refluxing of curcumin (2.0 mmol, 0.737 g) and CuSO_4_·5H_2_O (1.0 mmol, 0.249 g) in ethanol (20 ml) for 6 h and a dark green precipitate was obtained by this reaction. The obtained solid residue was isolated by filtration and washed by cold methanol and dried at air. Yield: 70%. *Anal*. Calcd. for C_42_H_38_CuO_12_: C, 63.19; H, 4.80; Cu, 7.96%. Found: C, 63.59; H, 4.65; Cu, 8.07%. FT-IR (KBr, cm^−1^): 3505 (br, m); 3423 (m); 2924 (w); 1624 (m); 1602 (m); 1509 (br, s); 1465 (w); 1452 (w); 1425 (m); 1280 (m); 1265 (m); 1207 (w); 1166 (m); 1112 (w); 968 (w); 817 (w); 540 (m); 446 (m); 438 (m); 423 (m); 417 (m); 412 (m).

#### Synthesis of Zn-CUR

The Zn-CUR complex was synthesized by a similar method used for the preparation of Cu-CUR. Zinc(II) chloride was used as the metal slat and a red–orange precipitate was obtained after refluxing the mixture for 6 h which was isolated by filtration and washed with cold ethanol. Yield: 69%. *Anal*. Calcd. for C_44_H_44_O_13_Zn: C, 62.45; H, 5.24; Zn, 7.73%. Found: C, 62.57; H, 5.16; Zn, 7.56%. FT-IR (KBr, cm^−1^): 3612 (br, w); 3531 (br, m); 3420 (br, m); 2937 (w); 2833 (w); 1625 (s); 1596 (s); 1562 (m); 1505 (vs); 1467 (m); 1428 (s); 1405 (s); 1289 (s); 1276 (s); 1220 (m); 1202 (m); 1157 (m); 1124 (m); 1026 (m); 985 (m); 973 (m); 954 (m); 839 (m); 817 (m); 602 (w); 579 (w); 549.61 (w); 468 (m).

#### Synthesis of Fe-CUR

The Fe-CUR complex was synthesized by refluxing of curcumin (3.0 mmol, 1.105 g) and Fe(NO_3_)_3_∙9H_2_O (1.0 mmol, 0.242 g) according the procedure described above. Yield: 65%. *Anal*. Calcd. for C_63_H_57_FeO_18_: C, 65.35; H, 4.96; Fe, 4.82%. Found: C, 66.01; H, 4.89; Fe, 4.75%. FT-IR (KBr, cm^−1^): 3512 (m, br); 3482 (m, br); 3450 (m, br); 3305; 3270; 3247; 3216; 2923 (w, br); 2851 (m, br); 1626 (s); 1602 (m), 1589; 1572; 1561; 1552 (m); 1546; 1510 (s); 1466; 1451; 1429.47; 1383; 1280 (m); 1234; 1206 (m); 1183; 1154 (m); 1123; 1026 (m); 977 (w); 965; 856; 815; 808; 669; 602; 541; 513.

### Determination of minimum inhibitory concentration

The broth microdilution method was used to determine the minimum inhibitory concentration (MIC) of metal-CUR complexes (Cu-CUR, Zn-CUR, Fe-CUR) and free-CUR (Bahari et al. 2017). Twofold serial dilutions of compounds (1000, 500, 250, 125, 62.5, 31.25, 15.6, 7.8, 3.9, 1.9 µg/ml) were prepared and then inoculated with PAO_1_ overnight culture containing 5 × 10^6^ CFU/ml and incubated at 37 °C for 24 h. Minimum inhibitory concentration was calculated as the lowest concentration that inhibited the organism’s visible growth.

### Cell growth analysis

The growth of treated and untreated *P. aeruginosa* PAO1 was measured by broth microdilution method (Bahari et al. 2017). Lysogeny broth containing different concentrations of free-CUR (1.9–500 µg/ml) and metal-CUR complexes (1.9–250 µg/ml) were inoculated with overnight culture of PAO1 and incubated at 37 °C for 16 h. Optical density of treated and untreated cultures was measured and the percentage of cell growth calculated as follow: OD_630_ (treated PAO_1_)/OD_630_ (untreated PAO_1_) × 100.

### Biosensor bioassay

To demonstrate the anti-QS activity of metal-CUR complexes (Cu-CUR, Zn-CUR, Fe-CUR) and free-CUR, the well diffusion was performed using the reporter strain *C. violaceum* CV026 (McClean et al. 1998). Fifty microliters of compounds at the concentrations of 1/4 and 1/16 MIC were loaded onto wells on the surface of *C. violaceum* CV026 inoculated Lysogeny agar plates supplemented with 20 µM of C6-HSL and incubated at 30 °C for 24 h. The presence of a pigmentless zone of viable cells around the well was considered as QS inhibition.

### Effects of biofilm formation

The effect of metal-CUR complexes (Cu-CUR, Zn-CUR, Fe-CUR) and free-CUR on the biofilm formation of *P. aeruginosa* PAO_1_ was assessed using microtiter plate method (Bahari et al. 2017). Lysogeny broth with and without sub-MIC concentrations (1/4 and 1/16 MIC) of compounds were inoculated with overnight cultures of PAO_1_ and incubated at 37 °C. After 24 h of incubation, biofilms were washed three times with sterile PBS, fixed with 150 µl of 99% (v/v) methanol and stained with 0.2% (w/v) crystal violet (HiMedia, India). Finally, 33% (v/v) glacial acetic acid was used to solubilize crystal violet. The absorbance of treated and untreated samples was measured at OD_590_ nm. The percentage of biofilm inhibition was calculated as follow: (1- OD590 of treated PAO_1_/OD590 of untreated PAO_1_) × 100. Reported values are the mean of three measurements.

### Motility assays

Swarming and twitching motilities were assessed on agar plates with or without a sub-MIC concentration of metal-CUR complexes (Cu-CUR, Zn-CUR, Fe-CUR) and free-CUR as described previously (El-Mowafy et al. 2014).

### Effects on alginate production

The effect of metal-CUR complexes (Cu-CUR, Zn-CUR, Fe-CUR) and free-CUR on the alginate production of *P. aeruginosa* PAO1 was assessed according to previous studies (Hoffmann et al. 2007; Manner and Fallarero 2018). Briefly, 5 ml of Lysogeny broth with and without sub-MIC concentrations (1/4 and 1/16 MIC) of compounds were inoculated with 500 µl of PAO1 suspension (1 × 10^8^ CFU/ml) and incubated at 37 °C. After 24 h of incubation, 1 ml of culture was centrifuged at 12,000 rpm for 30 min and the supernatant maintained at 80 °C for 30 min. The centrifuged supernatant was precipitated with ice-chilled ethanol 99% (v/v) at 4 °C for 2 h and mixed with 1 ml of sterile saline (0.9%). Then, one ml of borate sulfuric acid reagent (100 mM H_3_BO_3_ in concentrated H_2_SO_4_) and 34 µl of carbazole reagent (0.1% in ethanol) were added to 118 µl of sample on ice. The mixture was heated for 30 min at 55 ℃ and the absorbance at OD530 nm was measured.

### Effects on pyocyanin production and hydrogen peroxide susceptibility

The effect of metal-CUR complexes (Cu-CUR, Zn-CUR, Fe-CUR) and free-CUR on pyocyanin production and hydrogen peroxide (H_2_O_2_) susceptibility of *P. aeruginosa* PAO1 was assessed according to previous studies (Essar et al. 1990; Hoffmann et al. 2007; He, Hwang et al. 2014). For assay of pyocyanin production, treated (1/4 and 1/16 MIC) and untreated Lysogeny broth were inoculated with PAO1 suspension (1 × 10^8^ CFU/ml) and incubated at 37 ℃ for 24 h. Five ml of cell free supernatant was mixed with 3 ml of chloroform. One ml of HCl (0.2 M in distilled water) was added to extracted phase with chloroform. After centrifugation at 12,000 rpm for 10 min, the absorbance of red phase was measured at OD_520_ nm. The pyocyanin concentration was determined as µg/ml = (OD_520_ × 17.072). In order to determine hydrogen peroxide susceptibility, treated (1/4 and 1/16 MIC) and untreated Lysogeny broth were inoculated with PAO1 suspension (1 × 10^8^ CFU/ml) and incubated at 37 °C for 24 h. After centrifugation at 5000 rpm for 10 min, 100 µl of the supernatant were poured onto Lysogeny agar plates and a filter paper saturated with H_2_O_2_ (10%, v/v) was placed in the center of plates. The inhibition zone diameter was measured and reported in mm after 24 h of incubation at 37 °C.

### RNA extraction and cDNA synthesis

Total RNA of cultivated PAO_1_ with and without sub-MIC concentrations (1/2 and 1/16 MIC) of metal-CUR complexes (Cu-CUR, Zn-CUR, Fe-CUR) and free-CUR was extracted using EZ-10 Spin Column Total RNA Miniprep Super Kit (Bio Basic, Canada) with on-column DNaseI digestion (Bio Basic, Canada) according to the kit handbook. The concentration and purity of extracted RNA samples were determined using a NanoDrop Spectrophotometer (ND-1000, Nano-Drop Technologies, Wilmington, DE). Complementary DNA (cDNA) was then synthesized using PrimeScript™ RT reagent Kit (Takara, Japan). Reverse transcription was performed in a reaction mixture with total volume of 10 µl containing 2 µl of 5X PrimeScript Buffer, 0.5 µl of PrimeScript RT Enzyme Mix I, 0.5 µl of Random 6 mers (100 μM), 500 ng of RNA and RNase free water to complete the volume. The reactions were incubated at 37 °C for 30 min, 85 °C for 5 s and 4 °C for 10 min.

### Quantitative real time PCR

The effect of sub-MIC concentrations of metal-CUR complexes (Cu-CUR, Zn-CUR, Fe-CUR) and free-CUR on expression of quorum sensing genes *lasI* and *lasR* in PAO_1_ was evaluated as described previously (Bahari et al. 2017). Real time PCR was performed in a reaction mixture with total volume of 20 µl containing 10 µl of TB Green Premix Ex Taq (Takara, Japan), 0.4 µl of each primer (10 µM), 0.4 µl of ROX Reference Dye (50X), 1 µl of cDNA (100 ng) and 7.8 µl sterile purified water to complete the volume. Assays were performed in triplicate with an Applied Biosystems StepOnePlus™ Real-Time PCR System. The expression of the target genes was normalized to the expression of reference gene *oprL* (encoding the outer membrane protein) (Joly et al. 2005). Melting curve analysis demonstrated that the accumulation of TB Green-bound DNA was target gene specific. The no template control (NTC) and no reverse transcriptase control (no-RT) were included in all experiments. The expression of cultures grown in the presence of sub-MIC concentrations of compound were compared with untreated cultures and the data were analysed using the 2^−ΔΔCt^ method (McClean et al. 1998).

### Statistical analysis

The SPSS version 17.0 software (SPSS, Inc., Chicago, IL) was used to analyze data. Results were expressed as means and standard deviations of ∆C_t_ values. The One Way ANOVA was used to determine the statistical significant differences between the means of groups. A *P* value of < 0.05 was considered significant.

## Results

### Synthesis and characterization of metal-CUR complexes

**Scheme 1:**
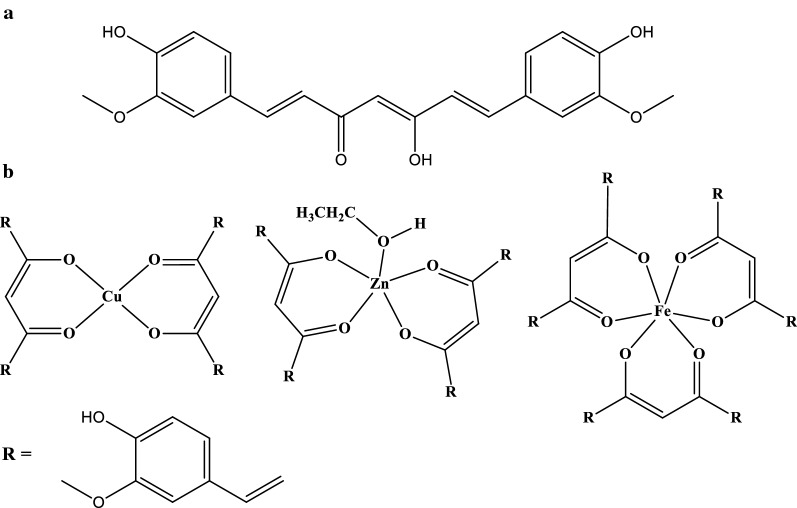
The molecular structures of the curcumin and Cu-CUR, Zn-CUR and Fe-CUR complexes

The metal-CUR complexes were synthesized by the reaction of curcumin with Cu(II), Zn(II) and Fe(III) salts in 1:2 (Cu and Zn) and 1:3 (Fe) metal: curcumin ratio in ethanol. The complexes were obtained as precipitates which were isolated by filtration and washed with ethanol. The molecular structures of products are shown in Scheme 1. The elemental analysis of the complexes and their FT-IR spectrum are in agreement with the proposed structures. In the FT-IR spectra of products the changes in the position of bands respect to the FT-IR spectrum of free curcumin confirms the coordination of curcumin to the metal ions. The band at about 1625 cm^−1^ in the FT-IR spectra of complexes is due to the presence of coordinated C=O to the metal ion. The bands at about 3400 cm^−1^ is related to the OH group and confirms the phenolic OH is not coordinated to the metal ions. The structures in Scheme 1 are also in agreement with the structure of similar products reported in CCDC database with curcumin or other beta-diketones with Cu(II), Zn(II) and Fe(III) ions. Considering CCDC database (Groom et al. 2016) indicates the Cu(II) ion stabilizes by coordination of two beta-diketone moiety with square planar geometry (Meza-Morales et al. 2019a) while Fe(III) ion forms complexes with three molecules of beta-diketones (Pousaneh et al. 2019). In the case of Zn(II), the complexes are stable with two beta-diketones and one or two solvent molecules also coordinate to the metal ion to generate five or six coordinated complexes, respectively (Aliaga-Alcalde et al. 2012). Considering elemental analysis confirms the formation of such structures in the case of Cu(II), Zn(II) and Fe(III) ions.

### Determination of MIC and cell growth

**Fig. 1:**
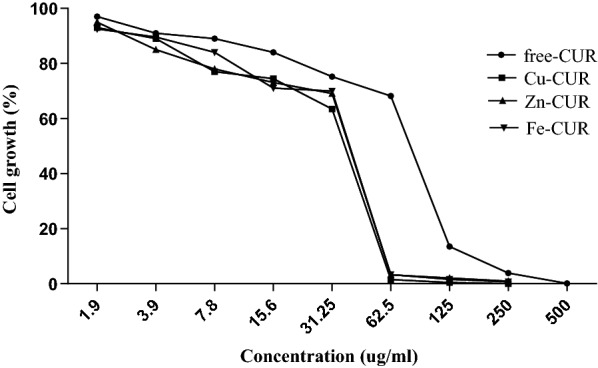
The effect of concentrations of 1.9 to 500 µg/ml of metal-CUR complexes (Cu-CUR, Zn-CUR, Fe-CUR) and free-CUR on cell growth of PAO1. The One Way ANOVA was used to determine the statistical significant differences between the means of groups

The MIC of metal-CUR complexes (Cu-CUR, Zn-CUR, Fe-CUR) and free-CUR against *P. aeruginosa* PAO_1_ was obtained 62.5 and 125 µg/ml respectively. The effect of different concentrations of metal-CUR complexes (Cu-CUR, Zn-CUR, Fe-CUR) and free-CUR on cell growth of PAO1 is shown in Fig. 1. The concentration of 125 µg/ml (1 × MIC) of free-CUR reduced the cell growth to 13.5% in comparison with untreated PAO_1_. However, Cu-CUR, Zn-CUR and Fe-CUR complexes at the concentration of 62.5 µg/ml (1 × MIC) significantly reduced the cell growth to 1.5%, 3.2% and 3.3%, respectively (*P *< 0.05). The cell growth was not significantly reduced at the concentrations of 1/16 MIC of compounds compared with untreated control.

### Biosensor bioassay

Metal-CUR complexes (Cu-CUR, Zn-CUR, Fe-CUR) and free-CUR showed anti-quorum sensing activity in *C. violaceum* CV026 biosensor bioassay. The concentrations of 1/4 and 1/16 MIC of free-CUR showed the pigmentless zones of 14 and 9 mm, respectively, indicating the violacein inhibition around the wells. Copper-CUR complex at the concentrations of 1/4 and 1/16 MIC showed pigmentless zones of 25 and 17 mm, respectively. Pigmentless zones were observed at the concentrations of 1/4 and 1/16 MIC of Fe-CUR (20 and 13 mm) and Zn-CUR (18 and 12 mm) complexes.

### Effects on biofilm formation

**Fig. 2:**
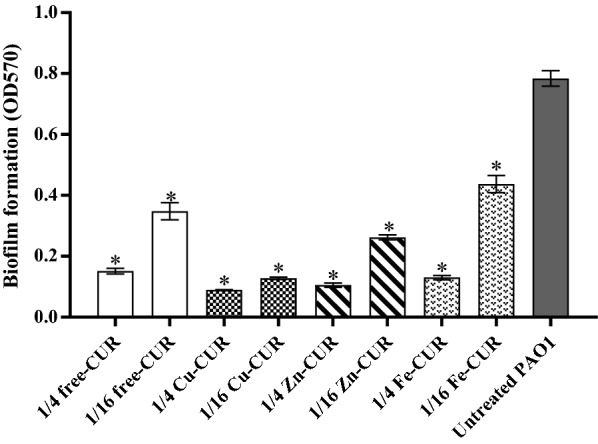
Effect of subinhibitory concentrations (1/4 and 1/16 MIC) of metal-CUR complexes (Cu-CUR, Zn-CUR, Fe-CUR) and free-CUR on biofilm formation. The One Way ANOVA was used to determine the statistical significant differences between the means of groups (*, significant, P < 0.05)

The biofilm formation was inhibited in the presence of metal-CUR complexes (Cu-CUR, Zn-CUR, Fe-CUR) and free-CUR between 45 and 90% (Fig. 2). The inhibitory effect was concentration dependent. Copper-CUR complex at the concentration of 1/4 MIC showed the greatest inhibitory effect on biofilm formation with 90% reduction (*P *< 0.001).

### Effects on motility

**Fig. 3:**
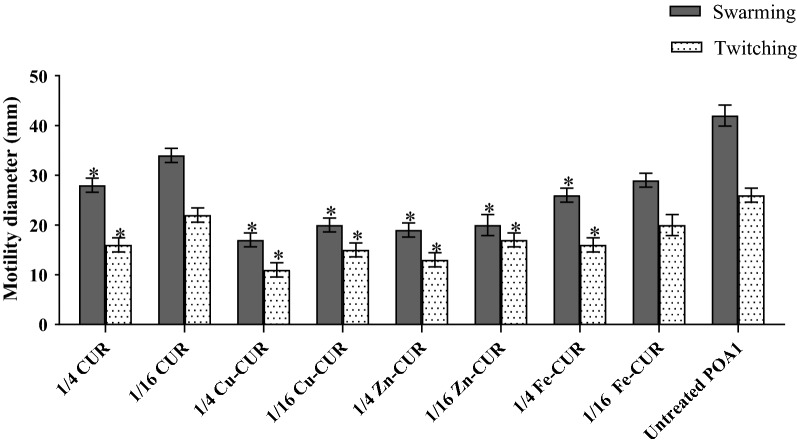
Effect of subinhibitory concentrations (1/4 and 1/16 MIC) of metal-CUR complexes (Cu-CUR, Zn-CUR, Fe-CUR) and free-CUR on the swarming and twitching motilities. The One Way ANOVA was used to determine the statistical significant differences between the means of groups (*, significant, P < 0.05)

The effect of metal-CUR complexes (Cu-CUR, Zn-CUR, Fe-CUR) and free-CUR on motility of PAO1 is shown in Fig. 3. There was a 38.5% decrease in twitching and 33.3% decrease in swarming motility in the presence of free-CUR at the concentration of 1/4 MIC. However, there was no significant effect on motility at 1/16 MIC of free-CUR. Copper-CUR complex at the concentration of 1/4 MIC significantly decreased motility and showed the greatest inhibitory effect on twitching and swarming motilities with 57.7% and 59.5% reduction, respectively (*P *< 0.05).

### Effects on alginate production

**Fig. 4:**
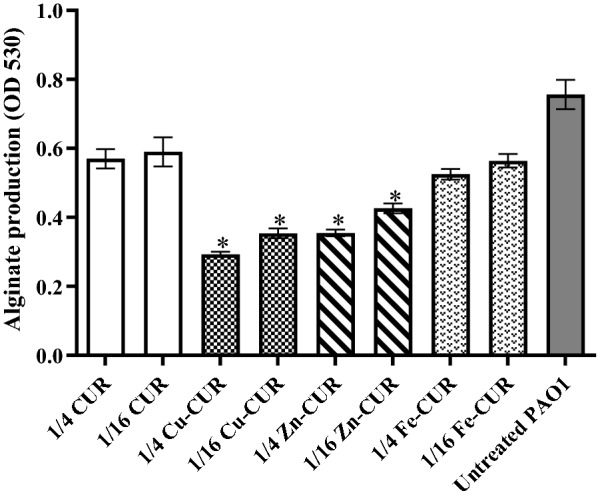
Effect of subinhibitory concentrations (1/4 and 1/16 MIC) of metal-CUR complexes (Cu-CUR, Zn-CUR, Fe-CUR) and free-CUR on the alginate production. The One Way ANOVA was used to determine the statistical significant differences between the means of groups (*, significant, P < 0.05)

The concentrations of 1/4 and 1/16 MIC of metal-CUR complexes (Cu-CUR, Zn-CUR, Fe-CUR) and free-CUR exhibited 21.3% to 62.6% reduction in alginate production compared with untreated PAO1 (Fig. 4). Copper-CUR and Zn-CUR complexes at the concentration of 1/4 MIC showed the greatest inhibitory effect on alginate production with 62.6% and 53.3% reduction, respectively (*P *< 0.05).

### Effects on pyocyanin production

**Fig. 5:**
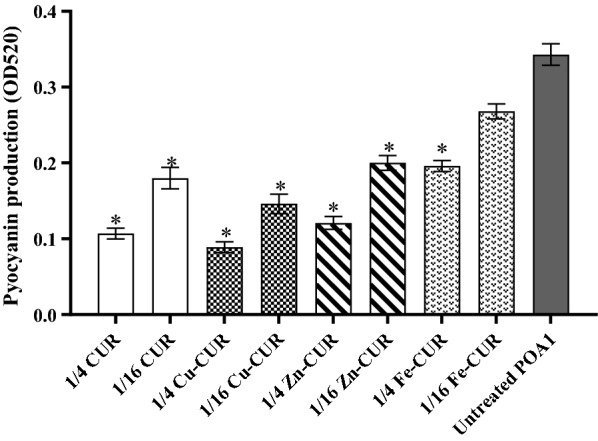
Effect of subinhibitory concentrations (1/4 and 1/16 MIC) of metal-CUR complexes (Cu-CUR, Zn-CUR, Fe-CUR) and free-CUR on pyocyanin production. The One Way ANOVA was used to determine the statistical significant differences between the means of groups (*, significant, P < 0.05)

The effect of metal-CUR complexes (Cu-CUR, Zn-CUR, Fe-CUR) and free-CUR on pyocyanin production is shown in Fig. 5. There was 26.5% to 76.5% decrease in pyocyanin production in the presence of these compounds. Copper-CUR complex at the concentration of 1/4 MIC significantly decreased pyocyanin production and showed the greatest inhibitory effect with 76.5% reduction (*P *< 0.05).

### Effects on hydrogen peroxide susceptibility

**Fig. 6:**
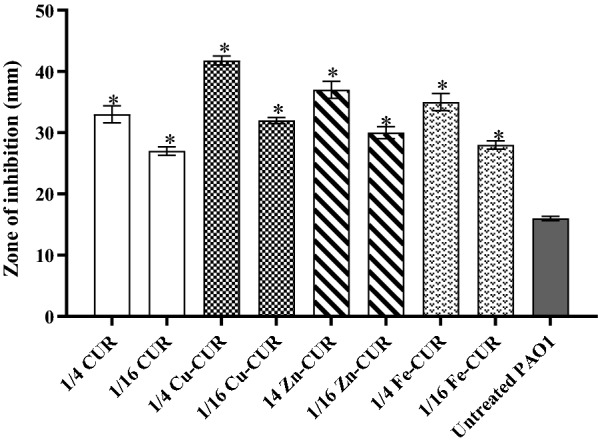
Effect of subinhibitory concentrations (1/2 and 1/16 MIC) of metal-CUR complexes (Cu-CUR, Zn-CUR, Fe-CUR) and free-CUR on H_2_O_2_ susceptibility. The One Way ANOVA was used to determine the statistical significant differences between the means of groups (*, significant, P < 0.05)

The concentrations of 1/4 MIC of metal-CUR complexes (Cu-CUR, Zn-CUR, Fe-CUR) and free-CUR significantly increased the sensivity of PAO1 to H_2_O_2_ (ranged from 1.7 to 2.7 fold) (*P *< 0.05). Copper-CUR complex at the concentration of 1/4 MIC showed the greatest sensivity to H_2_O_2_ by 2.6 fold (*P *< 0.05) (Fig. 6).

### Expression of quorum sensing regulated genes

**Fig. 7:**
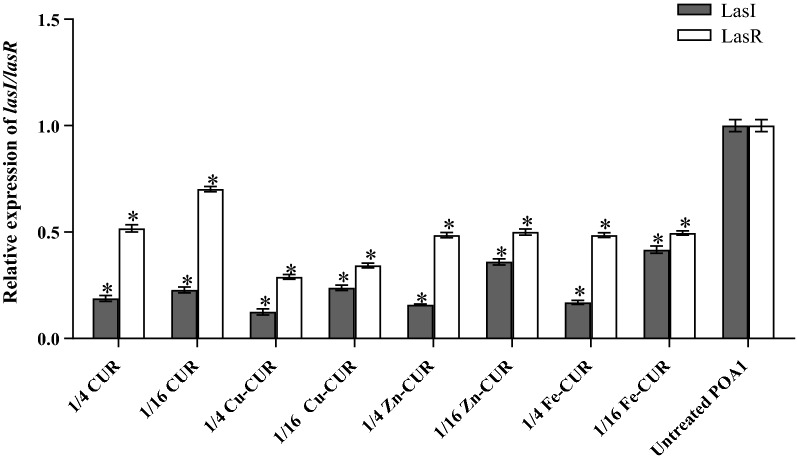
The subinhibitory concentrations (1/2and 1/16 MIC) of metal-CUR complexes (Cu-CUR, Zn-CUR, Fe-CUR) and free-CUR inhibited QS regulated genes in treated PAO_1_. The One Way ANOVA was used to determine the statistical significant differences between the means of groups (*, significant, P < 0.05)

Relative expression of quorum sensing regulated genes *lasI* and *lasR* was measured from calculated Ct values and standard curves. They showed the same melting profiles with no primer dimer formation. The standard curve of reference gene *oprL* and target genes *lasI* and *lasR* showed R2 values 0.99–0.97. Relative expression levels of treated cultures were compared with untreated cultures and the data were analyzed using the 2^−ΔΔCt^ method. Changes in expression level were shown in Fig. 7. Metal-CUR complexes (Cu-CUR, Zn-CUR, Fe-CUR) and free-CUR significantly repressed the expression of *lasI* and *lasR* between 30 and 88% relative to untreated PAO1 (*P *< 0.001). Copper-CUR complex at the concentrations of 1/4 MIC showed the greatest reduction in the expression levels of *lasI* (88%) and *lasR* (72%) compared with untreated control (*P *< 0.001).

## Discussion

Quorum sensing inhibitory activity of metal complexes is a promising strategy for treatment and control of bacterial infections (Behera et al. 2012; Wang et al. 2014; Ghosh et al. 2015; Meza-Morales et al. 2019b). Antimicrobial and special biological activities of metal complexes have been reported in previous studies (Bagchi et al. 2015; Syed et al. 2015; Girish et al. 2019; Tran Quang and Thao 2019). It has been found that the metal-curcumin complexes possess more antimicrobial activity against Gram-negative and Gram-positive bacteria than free ligands (Behera et al. 2012; Wang et al. 2014; Ghosh et al. 2015; Meza-Morales et al. 2019b). However, the effect of metal-curcumin complexes on *P. aeruginosa* QS system has not been reported. To our knowledge, this is the first study to demonstrate anti-quorum sensing activity of sub-MIC concentrations of metal-CUR complexes (Cu-CUR, Zn-CUR, Fe-CUR) against *P. aeruginosa* PAO1. According to our results, minimum inhibitory concentration of metal-CUR complexes and free-CUR against *P. aeruginosa* PAO_1_ was determined 62.5 and 125 µg/ml respectively. Bahari et al. (2017) also showed similar result on MIC value of free-CUR against *P. aeruginosa*. However, several previous studies have determined the antimicrobial activity of metal-curcumin complexes using Dick Diffusion method and the minimum inhibitory concentration of these compounds has not been investigated (Bagchi et al. 2015; Syed et al. 2015; Girish et al. 2019; Tran Quang and Thao 2019). Metal-CUR complexes and free-CUR decreased the cell growth of PAO1 at concentrations of 1 × MIC. But, the cell growth was not significantly reduced at concentrations of 1/4 and 1/16 MIC, proving that their anti-quorum sensing activities were achieved through inhibition of QS not by killing of cells. Similar results were reported by El-Mowafy et al. (2014) which showed that sub-MIC concentrations of aspirin significantly decreased the quorum sensing signals of *P. aeruginosa* without reduction in cell growth. Although we did not measure the anti-QS activity of metal-CUR complexes directly against PAO1, they indicated anti-QS activity in *C. violaceum* CV026. Free-CUR showed a concentration-dependent reduction in violacein production. However, Cu-CUR complex showed stronger anti-QS activity in comparison with other complexes and free-CUR. In agreement with our results, sub-MIC concentrations of natural and synthetic compounds such as furanone and its derivatives, quercetin, pyridoxal lactohydrazone, cinnamaldehyde and its derivatives, iberin, ajoene, catachin significantly inhibit QS signals and QS related virulence factors such as biofilm and motility (Tang and Zhang 2014; Gopu et al. 2015; Heidari et al. 2017; Manner and Fallarero 2018). The concentration of 31.2 µg/ml (1/4 MIC) of free-CUR significantly inhibited the biofilm formation in comparison with untreated PAO1. However, Cu-CUR complex at the concentration of 15.6 µg/ml (1/4 MIC) showed the greatest inhibitory effect on biofilm formation with 90% reduction. Significant reduction in biofilm production was also demonstrated at sub-MIC concentrations of curcumin in combination with azithromycin and gentamicin in our previous study (Bahari et al. 2017). In study conducted by Wang et al. (2014), ZnO nanoparticles inhibited *P. aeruginosa* biofilm formation and virulence factor production dose-dependently (Wang et al. 2014). Furthermore, ZnO/curcumin nanocomposites showed a significant reduction in virulence of *P. aeruginosa* via LasR-RhlR QS systems in comparison with curcumin and ZnO nanoparticles alone (Prateeksha Rao et al. 2019).

Swarming and twitching motilities in PAO1 treated with metal-CUR complexes or free-CUR were significantly impaired relative to untreated control. However, motility inhibition in the presence of metal-CUR complexes was higher compared with free-CUR. Copper-CUR complex at the concentration of 1/4 MIC showed the greatest inhibitory effect on twitching and swarming motilities with 57.7% and 59.5% reduction, respectively. These results are consistent with reports which showed that curcumin (Bahari et al. 2017), aspirin (El-Mowafy et al. 2014), pyridoxal lactohydrazone (Heidari et al. 2017) and tea polyphenols (Yin et al. 2015) inhibit swarming and twitching motilities in *P. aeruginosa.*

In our study, Cu-CUR complex at the concentration of 1/4 MIC showed the greatest inhibitory effect on alginate and pyocyanin production with 62.6% and 76.5% reduction, respectively. According to previous studies, reduced alginate production was observed at the concentrations of 1/4 MIC of pyridoxal lactohydrazone and quercetin compared with untreated control (Gopu et al. 2015; Heidari et al. 2017). Furthermore, the sub-MIC concentrations of aspirin (El-Mowafy et al. 2014), pyridoxal lactohydrazone (Heidari et al. 2017), marine oligosaccharides (He, Hwang et al. 2014) and cinnamaldehyde and salicylic acid (Ahmed et al. 2019) exhibited the inhibitory effects on pyocyanin production. We also confirmed that 1/4 MIC of Cu-CUR complex significantly made bacterial strains more susceptible to H_2_O_2_. Similar results were reported by He et al. that demonstrated the feasibility of attenuating the tolerance of *P. aeruginosa* to azithromycin by using marine oligosaccharides (He, Hwang et al. 2014).

Our study indicated that metal-CUR complexes and free-CUR significantly repressed the expression of *lasI* and *lasR* relative to untreated PAO1. Copper-CUR showed the greatest reduction in the expression levels of *lasI* and *lasR* at the concentration of 1/4 MIC. Considering the key role of quorum sensing in regulation of PAO1 virulence factors, we speculated that inhibition of these factors by Cu-CUR and other metal-CUR complexes is achieved through their effects on QS.

In conclusion, metal-CUR complexes (Cu-CUR, Zn-CUR, Fe-CUR) were synthesized and then characterized by spectroscopic methods. The effect of synthesized complexes on QS related virulence factors of *P. aeruginosa* PAO1 was also investigated. Our results indicate the potential of Cu-CUR complex at the concentration of 1/4 MIC to inhibit biofilm formation and QS related genes and virulence traits. Considering the biological effects of Cu-CUR complex and its inhibitory activity on virulence factors, it may be used as an effective compound for treatment and control of infections caused by *P. aeruginosa.*

## Data Availability

The supporting data for present findings presented here.
